# Nutritional interventions for osteoarthritis: targeting the metabolism-inflammation-oxidative stress axis—clinical evidence and translational practice

**DOI:** 10.3389/fnut.2025.1661136

**Published:** 2025-10-27

**Authors:** Yushan Wang, Zhiyan Cao, Yingjie Gao, Pengfei Shao, Shuai Gao, Mingjie Dong, Zui Tian, Yi Feng, Jiake Xu, Chuan Xiang

**Affiliations:** ^1^Department of Orthopedics, The Second Affiliated Hospital of Shanxi Medical University, Taiyuan, Shanxi, China; ^2^School of Biomedical Sciences, The University of Western Australia, Perth, WA, Australia; ^3^Shenzhen Institute of Advanced Technology Chinese Academy of Sciences, Shenzhen University of Advanced Technology, Shenzhen, China

**Keywords:** nutritional interventions, osteoarthritis, metabolism, inflammation, oxidative stress, gut-joint axis

## Abstract

Osteoarthritis (OA) is the most common chronic joint disease worldwide. Increasing studies have confirmed that obesity, metabolic status and gut microbiota imbalance can promote the occurrence of OA through the “metabolism-inflammation-oxidative stress” network, which is closely related to daily nutrition or dietary intake. The key nutrients with therapeutic effects mainly exert anti-inflammatory, anti-oxidative and chondro-protective effects. Among them, *ω*-3 polyunsaturated fatty acids and polyphenols are important components of anti-inflammatory diets, while collagen peptides, vitamin D, calcium, probiotics, glucosamine, chondroitin and hyaluronic acid are commonly used in clinical practice as important nutritional support treatments or preventive measures for OA to promote cartilage repair. In terms of dietary patterns, the Mediterranean diet (MD) rich in various nutrients can be used as the basic pattern for OA patients due to its anti-inflammatory and anti-oxidative properties and good clinical effects. Based on MD and evidence from clinical studies, this review constructs a four-level progressive nutritional plan for OA patients with the goals of relieving pain, delaying cartilage degeneration, improving function, and reducing the need for drugs and surgical intervention. We have also proposed customized nutritional management strategies for several special OA populations to reduce the occurrence of nutrition-related adverse events. Collectively, systematic nutritional intervention is expected to become the third major treatment alongside physical and drug therapy, enabling more OA patients to avoid adverse effects caused by repeated drug use and potential risks associated with surgery and prosthesis replacement.

## Introduction

Osteoarthritis (OA) is often misperceived as an inevitable wear-and-tear disease of aging. Yet, the startling reality is that metabolic and nutritional factors, many of which are modifiable, are now recognized as primary drivers of its pathogenesis, contributing to a global burden that afflicts over 300 million people (1). This redefinition of OA from a purely mechanical to a metabolic-inflammatory disorder opens transformative avenues for management, positioning nutritional intervention not merely as supportive care, but as a cornerstone of disease-modifying strategy.

OA stands as the most common chronic joint disease and a leading cause of disability globally (2–4). Its hallmark pathological features including cartilage degradation, subchondral bone sclerosis, osteophyte formation and synovial inflammation, are fueled by a complex interplay of inflammatory cascades, oxidative stress, and mechanical stress imbalance (5–7). While non-modifiable risks like age, gender, and genetics are well-known, emerging evidence underscores that obesity, metabolic syndrome, and gut dysbiosis are potent, modifiable risk factors, intricately linked to daily dietary patterns (8, 9).

Capitalizing on this understanding, nutritional interventions target OA through a multi-tiered approach: (1) Upstream regulation using anti-inflammatory nutrients (e.g., omega-3 fatty acids, polyphenols) to quell systemic inflammation and oxidative stress (10); (2) Downstream protection with cartilage-building substrates (e.g., collagen peptides, vitamin D) to enhance matrix integrity (11, 12); and (3) Systemic intervention through dietary modulations (e.g., weight loss, glycemic control) to ameliorate metabolic drivers and joint loading (13).

As the evidence base solidifies, the future of OA care lies in personalization, tailoring nutritional plans based on genetics, gut microbiota, and individual metabolic profiles. Therefore, this review will critically synthesize the pathophysiological links between nutrition and OA, evaluate the evidence for key nutrients and dietary patterns, and propose a framework for implementing personalized nutrition strategies to reshape the clinical management of this debilitating disease.

## The pathological association between nutrition and OA

### Obesity and OA: intertwined pathways of inflammation, mechanics, and diet

Obesity is a critical risk factor for OA, driven by a synergy of mechanical overload and adipose tissue-mediated systemic inflammation. Beyond simple biomechanics, where each 1 kg of weight gain multiplies knee joint load by 3–4 kg, accelerating cartilage wear and activating pathogenic pathways like Wnt/*β*-catenin, the endocrine function of fat is paramount (14, 15).

The pathological role of adipose tissue extends beyond mere energy storage. Pro-inflammatory adipokines (e.g., leptin, resistin) and adipose-derived exosomes (carrying miRNAs like miR-3074-5p) create a chronic inflammatory joint microenvironment by polarizing synovial macrophages toward an M1 phenotype and inhibiting anti-inflammatory adiponectin (16–18). This paradigm explains the high prevalence of OA in non-weight-bearing joints (e.g., hands) among obese individuals, a finding that cannot be attributed to mechanical load alone. Furthermore, obesity-related metabolic dysfunction exacerbates cartilage damage. Elevated palmitate induces chondrocyte apoptosis via endoplasmic reticulum stress (ERS), while a high-fat diet promotes the formation of advanced glycation end products (AGEs), which upregulate MMPs through the RAGE receptor (19–21).

Crucially, these pathways are fueled by specific dietary risk factors. The chronic consumption of a Western-style diet, which is rich in saturated fats, refined carbohydrates, and processed foods, directly promotes weight gain and establishes a state of meta-inflammation. This dietary pattern not only expands adipose tissue mass but also fundamentally alters its secretory profile, increasing the release of the very adipokines and exosomes that drive joint destruction.

However, a key counterargument persists. Not all obese individuals develop OA, and not all OA patients are obese. This suggests significant heterogeneity, likely mediated by genetic predispositions, specific adipose tissue distribution (e.g., visceral vs. subcutaneous), and other pathological factors.

### Oxidative stress and cartilage damage

Oxidative stress is also an important mechanism causing cartilage damage. Oxidative stress mainly damages cartilage homeostasis through excessive accumulation of reactive oxygen species (ROS). The specific mechanisms are as follows. (1) ROS directly attacks the mitochondria of chondrocytes, causing leakage of the electron transport chain (ETC) and further generating superoxide anion (O₂^−^), which forms a vicious cycle, inducing collapse of mitochondrial membrane potential and ATP synthesis disorder. (2) ROS activates NF-κB and MAPK signaling pathways, upregulating the expression of MMPs (MMP-13, MMP-3) and inflammatory factors (IL-1β, TNF-*α*), thereby accelerating the degradation of type II collagen (Col-II) and proteoglycans. (3) ROS induces DNA oxidative damage (such as formation of 8-OHdG) and lipid peroxidation (MDA increase), triggering chondrocyte apoptosis through Bax/Bcl-2 imbalance and activation of caspase-3. (4) Furthermore, the oxidative stress imbalance, caused by excessive ROS production, impairs the antioxidant defense system by directly damaging antioxidant enzymes (such as SOD and GPx) and depleting key antioxidant molecules like glutathione, leading to fragmentation of hyaluronic acid in the extracellular matrix and abnormal collagen cross-linking, thereby reducing the mechanical properties of cartilage (6, 22, 23). The implication of this self-perpetuating cycle is that oxidative stress is not merely a bystander but an active driver of OA progression, creating a feed-forward loop where inflammation begets more oxidative stress, and vice versa.

### The gut-joint axis: microbial dysbiosis and OA

Emerging evidence firmly establishes a link between gut microbiota dysbiosis and OA progression, primarily mediated by microbial metabolites via the “gut-joint axis.” The core protective mechanism involves short-chain fatty acids (SCFAs) like butyrate and propionate, produced from dietary fiber fermentation by commensals such as *Bacteroides* and *Faecalibacterium prausnitzii*. SCFAs activate GPCRs (GPR43/41) and inhibit histone deacetylases (HDAC), promoting regulatory T-cell (Treg) differentiation while suppressing Th17 cell polarization within the joint, thereby restoring immune homeostasis and reducing pro-inflammatory cytokine release. Concurrently, SCFAs enhance intestinal barrier integrity by upregulating tight junction proteins (e.g., occludin), which reduces systemic lipopolysaccharide (LPS) leakage and inhibits TLR4/NF-κB-driven synovial inflammation (24, 25). The significance of specific microbial shifts, such as an increased *Firmicutes*/*Bacteroidetes* ratio, extends beyond a mere descriptive biomarker. It implies a fundamental depletion of beneficial SCFA-producing taxa, creating a permissive environment for systemic inflammation and oxidative stress to accelerate chondrocyte apoptosis. Furthermore, the expansion of pathobionts like *Peptostreptococcus* can generate detrimental metabolites (e.g., indole derivatives), which directly upregulate cartilage-degrading MMP-13 (26–28).

However, several counterarguments and challenges temper the translational optimism. First, the causal direction in human studies remains ambiguous: it is unclear whether gut dysbiosis is a driver of OA or a consequence of its associated factors, such as reduced physical activity, diet changes, or chronic medication use. Second, the microbial landscape exhibits considerable inter-individual variation, suggesting that a universal “OA microbiome” signature may not exist, and interventions would likely require personalization (29). Finally, while rodent models demonstrate compelling proof-of-concept (e.g., SCFA supplementation ameliorates OA), the translatability of these findings to human physiology, with its greater complexity and longer disease course, is a significant hurdle that future research must overcome (30). Therefore, while maintaining gut microbiota stability represents a promising nutritional intervention strategy, its application necessitates a nuanced understanding of these complexities.

### The synergistic effect of nutritional-related metabolic syndrome on OA

Beyond obesity alone, the constellation of nutrition-related metabolic syndrome (MetS) components including insulin resistance, dyslipidemia, and hyperglycemia, synergistically exacerbates OA progression through interconnected pathways. This creates a deleterious metabolic milieu that accelerates joint damage. Hyperglycemia drives the accumulation of advanced glycation end products AGEs, which activate the RAGE/MAPK signaling axis, thereby inhibiting Col-II synthesis and promoting a catabolic state in chondrocytes (31). Concurrently, insulin resistance and resultant hyperinsulinemia impair chondrocyte homeostasis via the PI3K/Akt pathway, leading to suppressed FOXO1 transcription factor activity. This dual dysfunction reduces glucose uptake and antioxidant defense while paradoxically activating mTORC1 to promote cellular senescence (32, 33).

The critical implication of these findings is that OA should be reconceptualized, in a significant subset of patients, as a systemic metabolic disorder rather than a purely localized joint disease. This paradigm shift underscores that therapeutic strategies must target the underlying metabolic dysfunction, not just symptomatic pain relief.

However, a key counterargument challenges a direct causative role. The clinical correlation between MetS and OA is robust, yet not all individuals with MetS develop severe OA, and vice versa. This indicates substantial phenotypic heterogeneity and suggests that metabolic factors likely act as potent disease accelerants in genetically or biomechanically susceptible individuals, rather than being universal initiators (34). Furthermore, the relative contribution of each metabolic abnormality (e.g., hyperglycemia vs. dyslipidemia) to joint damage remains poorly defined, complicating the development of targeted nutritional interventions. Disentangling these individual effects represents a crucial future direction for personalized medicine in OA.

**Figure 1:**
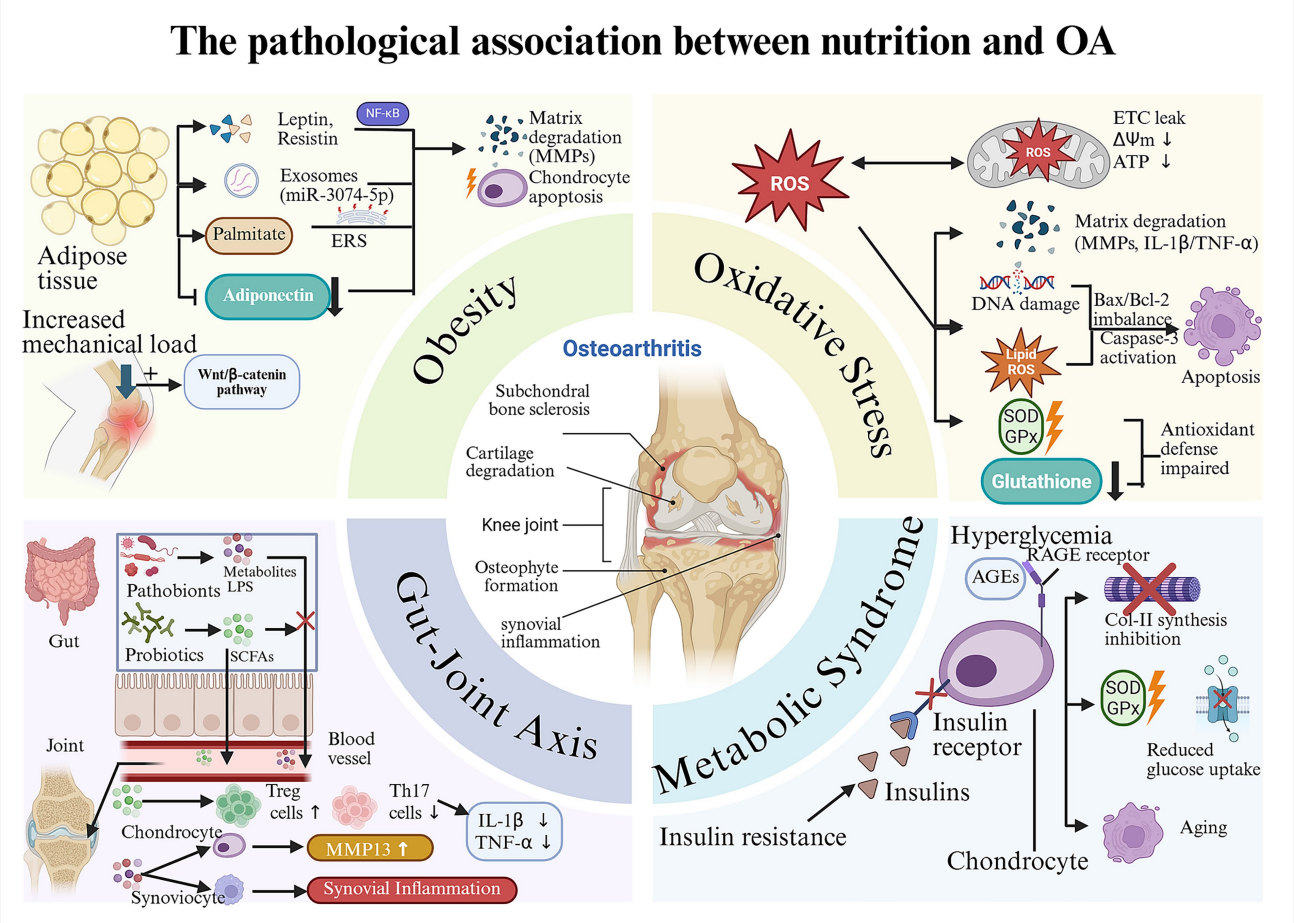
The pathological association between nutrition and OA: “metabolism-inflammation-oxidative stress” axis. Obesity: High-fat/high-sugar diets promote the proliferation and secretion profile alteration of adipose tissue, which further leads to the degradation of the cartilage matrix and the apoptosis of chondrocytes. The increased mechanical load caused by obesity can also accelerate cartilage wear. Oxidative stress: Unhealthy dietary habits such as high-fat/high-sugar diets can also cause cartilage damage by activating oxidative stress, which is specifically divided into the mitochondrial pathway, the inflammatory pathway, the apoptotic pathway and the antioxidant defense impairment pathway. Gut-joint axis: The imbalance between beneficial and harmful bacteria in the gut can lead to a decline in the defense capacity induced by SCFAs, subsequently causing a large amount of harmful substances (such as LPS) to enter the bloodstream. These harmful substances can cause inflammatory damage within the joint cavity. Metabolic Syndrome: Metabolic syndrome (taking insulin resistance and hyperglycemia as examples) can cause cartilage damage through interconnected pathways (the AGEs-RAGE pathway and various effects triggered by the dysfunction of insulin receptors).

In conclusion, OA pathogenesis is propelled by a metabolically dysregulated milieu, where obesity, oxidative stress, gut dysbiosis, and broader metabolic syndrome converge to create a self-perpetuating cycle of inflammation and tissue damage (Figure 1). Future research must therefore pivot toward integrated, multi-targeted nutritional strategies that simultaneously address these interconnected pathways, moving beyond single-nutrient approaches to achieve personalized OA management (35).

## Evidence-based effects of key nutrients

After understanding the pathological connection between nutrients and OA and discovering the significant role of the “metabolism-inflammation-oxidative stress” network in it, this study explores key nutrients that can prevent or treat OA through evidence-based medicine and reveals the mechanisms by which they exert preventive or therapeutic effects (Table 1).

**Table 1:** Therapeutic mechanisms and evidence-based effects of key nutrients associated with OA.

Types of nutrients	Therapeutic mechanisms	Follow-up duration	Clinical evidence (SMD^a^)		Level of evidence^b^
			Pain^c^	Function^c^	
Anti-inflammatory nutrients	Anti-inflammation				
ω-3 PUFAs	Inhibiting the NF-κB pathway and pro-inflammatory metabolites of arachidonic acid (29, 30)	<6 mo	−0.39 [−0.70, −0.09], 4 trials (33)	−0.33 [−0.77, 0.11], 3 trials (33)	Low
		≥6 mo	−0.23 [−0.44, −0.03], 5 trials (33)	−0.14 [−0.24, −0.05], 5 trials (33)	
Polyphenols	Inhibiting the NF-κB, MAPK and COX-2 pathways (35, 37)	4 wk	−0.04 [−0.25, 0.18], 1 trial (36)	−0.07 [−0.29, 0.14], 1 trial (36)	Low
		6 wk	−0.72 [−1.36, 0.07], 1 trial (36)	−0.91 [−1.56, −0.25], 1 trial (36)	
Chondroprotective nutrients	Cartilage repair				
Collagen peptides	Activating the TGF-β/Smad3 pathway and stimulating synthesis of Col-II and proteoglycans (41) synthesis	10–48 wk	−0.22 [−1.58, 0.13], 4 trials^d^ (43)	−0.62 [−5.77, 4.52], 4 trials^d^ (43)	Low
Vitamin D	Activating the VDR receptor, maintaining the bone density and inhibiting PTH (44, 46, 47)	2 years	−0.87 [−2.12, 0.38], 1 trial^d^ (51)	−3.11 [−6.52, 0.30], 1 trial^d^ (51)	Low
		3 years	−0.79 [−2.31 to 0.74], 1 trial^d^ (50)	−0.65 [−2.09 to 0.79], 1 trial^d^ (50)	
Antioxidant	Anti-oxidation				
Vitamin C	Eliminating ROS, inhibiting the NF-κB pathway and promoting synthesis of Col-II and proteoglycans (52, 53)	Not reported	Not reported	Not reported	Not reported
Vitamin E	Blocking the lipid peroxidation, inhibiting COX-2 and iNOS, and reducing PGE2 and NO (54–56)	≤ 2 years	−0.84 [−1.75 to 0.05], 5 trials (61)	−0.92 [−2.33 to 0.49], 5 trials (61)	Low
Selenium and Zinc	Removing hydrogen peroxide and lipid peroxides, inhibiting the NF-κB and MAPK pathway, and maintaining the redox balance (62–64, 69)	Not reported	Not reported	Not reported	Not reported
Bioactive spices	Anti-inflammation and anti-oxidation				
Ginger	Inhibiting COX-2 and LOX, reducing prostaglandin and leukotriene synthesis (89)	3-12wk (90)	−0.30 [−0.50 to −0.09], 5 trials (90)	Not reported	Low
Garlic	Upregulating glutathione for anti-oxidation and suppressing NF-κB for anti-inflammation (91)	Not reported	Not reported	Not reported	Not reported
Thyme	Scavenging free radicals and enhancing endogenous antioxidant defenses (92)	Not reported	Not reported	Not reported	Not reported
Controversial supplements					
Glucosamine and Chondroitin	Stimulating synthesis of proteoglycans and HA, and inhibiting the NF-κB pathway (73, 74)	≤ 2 years	−11.21 [−25.87, 3.45], 5 trials^d^ (77)	−7.23 [−14.47, 0.02], 5 trials^d^ (77)	Low
Hyaluronic Acid	Stimulating synthesis of endogenous HA, inhibiting the TLR4/NF-κB pathway and regulating SCFAs (82)	56 wk	−0.49 [−1.00, 0.02], 1 trial (85)	−1.18 [−1.73, −0.63], 1 trial (85)	Low
Probiotics					
*Lactobacillus casei Shirota*	Reduction in serum C-reactive protein (107)	6 mo	−0.93 [−1.61, −0.25], 1 trial (107)	−0.91 [−1.11, −0.71], 1 trial (107)	Low
*TCI633*	Metabolic production of HA (108)	12 wk	No significant difference, 1 trial (108)	No significant difference, 1 trial (108)	Low

a Standardized mean difference (SMD) is interpreted as 0.2 = small effect, 0.5 = medium effect and 0.8 = large effect.

b The level of evidence was assessed using the Grading of Recommendations, Assessment, Development, and Evaluations (GRADE) or specific criteria by the individual meta-analysis.

c For the outcome of pain, a negative SMD indicates less pain following the intervention. For the outcome of function, a negative SMD indicates improved function following the intervention.

d The data was used mean difference (MD) rather than SMD, and the WOMAC pain and function scores were adopted.

### Anti-inflammatory nutrients

#### ω-3 polyunsaturated fatty acids

**Figure 2:**
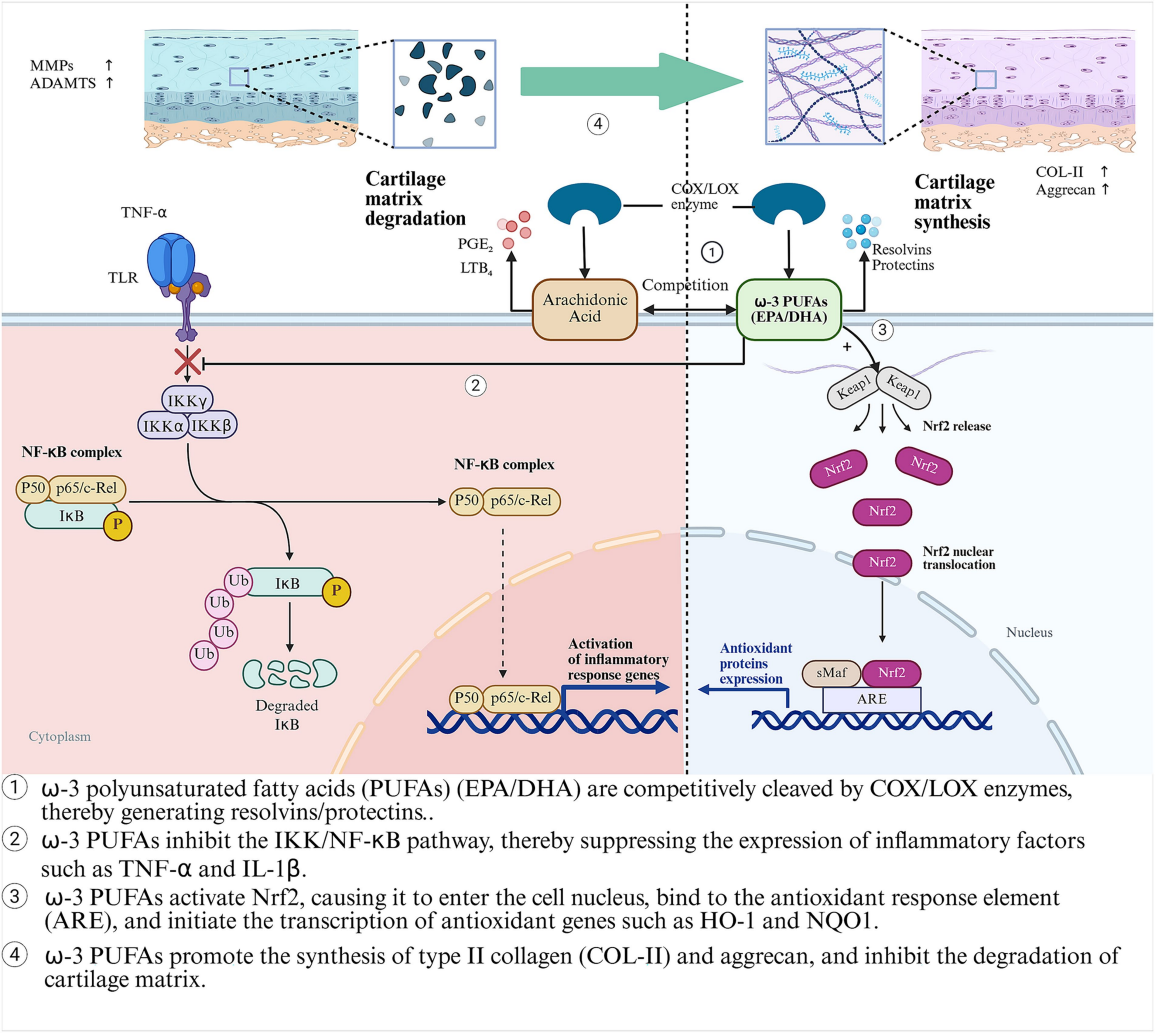
The anti-inflammatory, antioxidant and cartilage-protective mechanisms of *ω*-3 PUFAs (EPA/DHA) in OA.

ω-3 polyunsaturated fatty acids (ω-3 PUFAs) are a type of essential fatty acids with multiple double bonds. They are named because the first double bond is located on the third carbon atom from the methyl end. The main components include *α*-linolenic acid (ALA, from plants), eicosapentaenoic acid (EPA), and docosahexaenoic acid (DHA, from marine sources) (36). ALA is mainly found in flaxseeds, chia seeds, and walnuts. It needs to be converted into EPA and DHA through enzymatic reactions in the human body, but the conversion rate is relatively low (about 5–10%) (37). Fish oil (such as salmon and mackerel) and algae can directly provide bioactive forms of EPA and DHA (38). By inhibiting the NF-κB pathway and competitively antagonizing pro-inflammatory metabolites of arachidonic acid (such as PGE₂ and LTB₄), they exert anti-inflammatory and cartilage-protective effects (39, 40). Beyond anti-inflammation, *ω*-3 PUFAs also exhibit potent antioxidant properties by activating the Nrf2 signaling pathway (41). The combined anti-inflammatory and antioxidant effects converge to achieve chondroprotection (Figure 2). Flaxseeds, as a plant-based source of ALA, are more suitable for vegetarians. However, their anti-inflammatory effects are often inferior to direct supplementation of EPA/DHA due to the limited conversion efficiency (37). A randomized controlled trial (RCT) demonstrated that after 6 months of supplementation with 1.2 g/d of EPA + DHA for patients with knee OA, the WOMAC pain score decreased by 23% (*p* = 0.02), and the synovial inflammation assessed by MRI was relieved (42). A systematic review (including 9 RCTs) confirmed that *ω*-3 PUFAs could alleviate the pain symptoms of OA patients (*p* = 0.002) and improve joint function (SMD = −0.21) (43). The consistent symptom-modifying effect across trials strongly implies that ω-3 PUFAs are a viable dietary strategy for managing OA-related pain, particularly for individuals seeking or requiring adjunctive approaches to pharmacotherapy.

However, significant heterogeneity in EPA/DHA dosing (0.5–3 g/d) and ratios across studies, short trial durations (typically ≤6 months), and a lack of robust structural outcome data (e.g., joint space narrowing) have precluded their formal recommendation as a standard adjunctive treatment in clinical guidelines (44). This evidence gap highlights a critical disconnect between compelling biological mechanisms and the level of proof required for widespread clinical implementation. Therefore, future research must prioritize large-scale, long-term (≥2 years) RCTs employing standardized EPA/DHA formulations and doses targeted to achieve specific tissue or plasma levels. These studies should explicitly address whether *ω*-3 PUFAs offer disease-modifying effects by incorporating advanced imaging biomarkers of cartilage integrity. Ultimately, defining the optimal dosing regimen and identifying patient subgroups most likely to respond will be key to translating this promising nutritional intervention into personalized OA care.

#### Polyphenols

Polyphenols are a type of natural organic compounds widely present in plants. They possess multiple phenolic hydroxyl structures and are mainly classified into flavonoids, phenolic acids, curcuminoids, and stilbenes. Curcumin is the main active component in turmeric roots (belonging to the curcuminoid category). Studies have shown that it can reduce the release of pro-inflammatory factors by inhibiting the NF-κB and COX-2 pathways, and activate Nrf2 to enhance the activity of antioxidant enzymes (45). Even more, some polyphenols can act as plant estrogens or SERMs to alleviate the cartilage degeneration associated with menopausal OA (46). A double-blind RCT showed that after 8 weeks of oral administration of 1.5 g/d curcumin (containing piperine) to patients with knee OA, the WOMAC pain score significantly decreased (*p* = 0.03). The effect was comparable to that of ibuprofen and was safer (47). Resveratrol is a type of stilbenes polyphenol that can be found in grape skins, berries, and peanuts. It inhibits the NF-κB and MAPK signaling pathways by activating the SIRT1 deacetylase, reduces ROS production, and delays chondrocyte apoptosis (48). Clinical studies have shown that a 500 mg daily dose of resveratrol for 3 months can significantly reduce the VAS score, but the improvement in joint structure is limited (49).

The compelling safety profile of these polyphenols, especially when compared to non-steroidal anti-inflammatory drugs (NSAIDs), implies their prime utility as long-term adjunctive therapies or for individuals intolerant to conventional pharmacotherapy. However, a central counterargument revolves around the issue of low oral bioavailability, particularly for curcumin. The promising effects observed *in vitro* often require sophisticated formulations (e.g., with piperine, nanoparticles) to be partially translated in humans, raising questions about the efficacy of simple dietary intake or standard extracts (50). Consequently, future research directions must extend beyond efficacy confirmation. The priority lies in optimizing delivery systems to enhance bioavailability and conducting longer-term trials with structural outcomes as primary endpoints. Furthermore, exploring synergistic effects, either between different polyphenols or with conventional treatments, and identifying biomarkers to predict individual responses are crucial steps toward personalized nutrition strategies for OA.

### Chondro-protective nutrients

#### Collagen peptide

Collagen peptides are small molecular fragments of collagen (with a molecular weight of approximately 2–5 kDa) obtained through enzymatic processing. They mainly come from the cartilage, bones, and skin of cows, fish, or chickens, and have high bioavailability. The potential mechanisms of collagen peptides in the prevention or treatment of OA are as follows. (1) Stimulate chondrocytes to synthesize Col-II and proteoglycans, while inhibiting the activities of MMP-13 and ADAMTS-5, and reducing matrix degradation. (2) Promote cartilage repair by activating the TGF-*β*/Smad3 signaling pathway. (3) Inhibit the release of synovial inflammatory factors (51). Although there are not many studies on the relevant mechanisms, collagen peptides have still gained popularity among some patients with OA. A RCT showed that daily supplementation with 10.78 g of collagen peptide (COLLinstant^®^) for 6 months could significantly reduce the VAS pain score of knee OA patients (an average reduction of 43.6%, *p* < 0.001) and improve joint mobility (*p* < 0.02) (52). A meta-analysis involving 5 RCTs (with a total of 519 participants) indicated that the collagen peptide group showed significant superiority over the placebo group in terms of pain relief (WMD: −16.57, *p* < 0.001) and stiffness improvement (−0.41, *p* = 0.01), but the long-term impact on joint function still requires further verification (53). The consistent symptom-modifying effect, coupled with an excellent safety profile, positions collagen peptides as a well-tolerated adjunct to core OA management strategies like exercise and anti-inflammatory diets. However, a key counterargument hinges on the lack of robust evidence for disease-modifying effects. Most trials are of short duration and utilize varied sources and doses of collagen peptides (e.g., 8–12 g/d), and crucially, they lack convincing imaging data demonstrating a halt in cartilage loss or joint space narrowing (52). This suggests that while collagen peptides are effective for symptom control, their ability to structurally alter OA progression remains an open and critical question. Therefore, future research must prioritize long-term (≥2-year) RCTs with standardized, well-characterized preparations, using quantitative MRI biomarkers of cartilage volume or composition as primary endpoints. Additionally, exploring potential synergies with other anabolic agents (e.g., vitamin D) and identifying patient subgroups most likely to respond (e.g., based on baseline collagen turnover markers) will be essential for advancing personalized nutrition protocols in OA.

#### Vitamin D and calcium

Vitamin D (a fat-soluble vitamin) and calcium (an essential mineral) are crucial nutrients for maintaining bone and joint health. Vitamin D, obtained through skin synthesis and dietary sources (e.g., fatty fish, fortified foods), must undergo a two-step hydroxylation in the liver and kidneys to become its active form, 1,25-dihydroxyvitamin D₃ [1,25(OH)₂D₃]. Crucially, the enzymes facilitating this activation process are magnesium-dependent, underscoring the mineral’s essential role in vitamin D metabolism (54). Calcium is mainly found in dairy products, dark green vegetables and legumes. It is the main component of bones and teeth, and also participates in muscle contraction and nerve signal transmission (55). Vitamin D and calcium exert protective effects on OA by jointly regulating the bone-cartilage homeostasis. (1) Vitamin D, by activating the VDR receptor, inhibits the expression of inflammatory factors in chondrocytes and reduces the activity of MMP-13, while promoting the synthesis of Col-II (56). (2) Calcium maintains the bone density beneath the cartilage and prevents joint mechanical imbalance caused by abnormal bone remodeling. (3) Active vitamin D enhances intestinal calcium absorption (57). The two jointly inhibit the increase of parathyroid hormone (PTH), and reduce the risk of bone resorption and cartilage calcification (58). Clinical studies have shown that patients with OA who have a serum 25(OH)D_3_ level of less than 15 ng/mL experienced significantly increased pain, especially in male patients (*p* ≤ 0.003) (59). And the risk of OA progression has increased by more than twice (OR: 2.3; 95% CI: 1.1, 4.5) (60). Therefore, many clinical doctors recommend that patients with OA take appropriate supplements of vitamin D and calcium. The current recommended dosage for patients with OA is 1,000–2000 IU of vitamin D per day to maintain serum 25(OH)D_3_ at 30 ng/mL or higher (61, 62), and 1,000 mg of calcium per day to optimize bone and cartilage protection (63). However, many studies have found that supplementation of 2000 IU + 1,000 mg/d did not improve the symptoms (pain, stiffness, and limited mobility) of OA or delay the progression of the disease within a certain period (2–3 years) (64, 65). It can be seen that there is heterogeneity in the effect of vitamin D and calcium supplementation on symptom improvement, which may be partly explained by variable magnesium status among participants and the different follow-up time. Therefore, long-term RCTs and precise baseline control are necessary for studying the long-term efficacy and complications of vitamin D and calcium.

### Antioxidant

#### Vitamin C and E

Vitamin C (ascorbic acid) and vitamin E (tocopherol) are two crucial antioxidant nutrients that protect cartilage from oxidative damage by water-soluble and lipid-soluble status, respectively. Vitamin C is widely found in citrus fruits and green leafy vegetables. As a cofactor of proline hydroxylase, it directly promotes the synthesis of Col-II and proteoglycans (66). And by eliminating ROS and inhibiting the NF-κB pathway, it reduces mitochondrial oxidative damage in chondrocytes and the expression of inflammatory factors such as MMP-13 (67). Vitamin E, as a fat-soluble antioxidant, is mainly found in nuts and seed oils. It can protect the integrity of cell membranes by blocking the lipid peroxidation chain reaction (68), and can also inhibit activities of COX-2 and iNOS and reduce the production of PGE2 and NO to alleviate synovial inflammation (69, 70). Clinical studies have shown that high dietary intake of vitamin C (≥ 200 mg/d) can inhibit the progression of OA (OR = 0.3, 95% CI: 0.1–0.6) and reduce knee joint pain (OR = 0.3, 95% CI: 0.1–0.8), but it has no significant effect on preventing the occurrence of OA (71). On the contrary, Peregoy et al. proposed a different conclusion. They believed that supplementing vitamin C had certain positive significance for preventing OA, but it cannot inhibit the progression of OA (72). At present, there is still controversy regarding the effect of vitamin C on OA. Therefore, in clinical practice, most doctors do not emphasize the therapeutic significance of vitamin C supplementation for OA. Studies have shown that combined supplementation of vitamin C (1 gram twice daily) and E (100 mg three times daily) can improve the pain score of OA patients (73). The combined application can significantly enhance the antioxidative performance compared to single use. However, the long-term risks and therapeutic effects of the combined application still need to be further evaluated due to the lack of relevant RCTs. But studies have shown that the long-term use of vitamin E should be carefully considered. It may increase the risk of bleeding by 58% (74), and the therapeutic effect is not significant (75).

#### Selenium and zinc

Selenium and zinc are essential trace elements for the human body. Selenium is incorporated into glutathione peroxidase (GPx) in the form of selenocysteine, which removes hydrogen peroxide (H₂O₂) and lipid peroxides (such as MDA), reducing oxidative damage to chondrocytes. At the same time, it inhibits the NF-κB pathway and reduces the expression of IL-1β and MMP-13 to protect Col-II. It can also maintain the redox balance of chondrocytes through sequestin P (76–78). The dietary sources of selenium include Brazil nuts, seafood and whole grains. A Cross-Sectional Study showed that patients with lower serum selenium levels (<100 micrograms/L) were more likely to develop OA (OR ≥ 1.24), and there was a dose–response relationship (*p* = 0.005) (79). The result of a RCT demonstrated that daily supplementation with 200 μg of selenium (selenium-containing methionine) for 12 weeks had a certain positive effect in reducing the ESR level of RA patients [the effect size (95% CI) for ESR: 0.38 (−0.14, 0.89)], but it had no significant effect on reducing the serum CRP level (*p* = 0.05) (80). Although there are very few RCTs on the application of selenium in OA treatment, selenium supplements are not currently recommended for OA treatment considering the potential adverse effects of long-term use (5 years) (81). Appropriate food intake is supported [55–200 mg/d (maximum 400 mg/d)] (82). Zinc, acting as a cofactor for superoxide dismutase (SOD) and metallothionein (MT), neutralizes superoxide anions (O₂^−^) and inhibits MAPK inflammatory pathways (83), which helps protect the mitochondrial function of chondrocytes and reduces the degradation of cartilage matrix. It is commonly found in red meat, shellfish and beans. Although antioxidative and anti-inflammatory properties of zinc are more beneficial for the prevention and alleviation of OA theoretically, in fact, current mainstream clinical studies suggested that zinc supplementation and high serum concentrations were more likely to increase the risk of OA [*p* < 0.001, OR 95% CI = 1.044 (1.021–1.067)] (84, 85). The researchers believed that both zinc deficiency and excess were detrimental to bone health and immune function, and thus can accelerate the progression of OA (86). However, there is still a lack of sufficient evidence regarding the optimal concentration range of zinc and its actual therapeutic effect. Therefore, zinc supplementation is not recommended as a feasible treatment for OA. The dietary intake should be maintained at the recommended levels of 8–11 mg per day for adults (87), with a tolerable upper intake level of 40 mg per day to avoid potential toxicity (88).

### Bioactive spices

Beyond isolated nutrients, common culinary spices such as ginger, garlic, and thyme are rich sources of bioactive compounds with demonstrated efficacy in mitigating OA-related pathways. Their pleiotropic effects align perfectly with the metabolism-inflammation-oxidative stress network.

#### Ginger and its bioactive derivatives

The primary bioactive components, gingerols and shogaols, exhibit potent anti-inflammatory activity by inhibiting COX-2 and lipoxygenase (LOX), thereby reducing prostaglandin and leukotriene synthesis (89). A meta-analysis of randomized controlled trials confirms that ginger extract supplementation confers a modest but statistically significant reduction in knee pain and stiffness compared to placebo, with an effect size comparable to some conventional analgesics (90). The implication of these findings is that ginger represents a viable complementary strategy for OA symptom management, potentially enabling a reduction in conventional analgesic use. However, counterarguments highlight the heterogeneity in ginger preparation (dose, extract type) across studies and its moderate effect size, which may not be clinically meaningful for all patients. Gastrointestinal discomfort is also a noted side effect. Future research should prioritize standardized, high-quality extracts and focus on defining which patient phenotypes respond best to ginger intervention. Crucially, investigations into whether its anti-inflammatory effects translate to long-term disease modification beyond symptomatic relief are warranted.

#### Garlic and its organosulfur compounds

Garlic, a widely used culinary herb, has been recognized for its medicinal properties, primarily attributed to organosulfur compounds like allicin, diallyl disulfide (DADS), and S-allyl cysteine (SAC). These bioactive constituents mediate antioxidant effects by upregulating cellular glutathione levels and exhibit anti-inflammatory activity through suppression of the NF-κB signaling pathway, thereby potentially protecting chondrocytes from inflammatory and oxidative stress (91). While preclinical evidence for these mechanisms is robust, direct high-quality clinical evidence specifically for OA remains limited. A few pilot studies and investigations in related conditions, such as rheumatoid arthritis, suggest beneficial effects on inflammatory markers, but large-scale, long-term RCTs in OA populations are lacking. The implication of these mechanistic findings is that garlic or its standardized extracts could serve as a dietary component for mitigating OA pathophysiology. However, a significant counterargument revolves around the bioavailability and stability of allicin, which is highly volatile and rapidly metabolized. This raises questions about the consistency and efficacy of common garlic preparations. Future research must focus on employing well-characterized, stable garlic extracts with defined sulfur compound profiles in rigorous clinical trials. Furthermore, exploring potential synergistic effects of garlic with other nutraceuticals (e.g., ginger, turmeric) within the “Metabolism-Inflammation-Oxidative Stress” network represents a promising direction for developing multi-targeted dietary interventions.

#### Thyme and its bioactive monoterpenes

Thyme, a culinary and medicinal herb from the Lamiaceae family, is rich in bioactive monoterpenes, primarily thymol and carvacrol. These compounds exhibit potent antioxidant activity by directly scavenging free radicals and enhancing endogenous antioxidant defenses (92), which could help mitigate the oxidative stress and catabolic processes central to OA progression. However, a major counterargument is the significant gap between *in vitro* findings and the scarcity of *in vivo* or clinical evidence for OA. Challenges include the bioavailability of orally administered compounds and the translation of effective in vitro doses to practical human intake. Future research should prioritize well-designed animal studies to confirm efficacy in a whole-organism context. If promising, subsequent clinical trials should utilize standardized thyme extracts with defined chemical profiles to evaluate their effects on OA symptoms and structural progression, potentially as part of a broader dietary approach.

### Controversial supplements

#### Glucosamine and chondroitin

Glucosamine and Chondroitin are important components of cartilage matrix. Glucosamine, as an amino sugar precursor, has been reported by some studies to stimulate the synthesis of proteoglycans and hyaluronic acid (HA) by chondrocytes, inhibit the activities of MMP-3 and ADAMTS-5, and reduce the degradation of cartilage matrix (93). Chondroitin sulfate reduces the release of IL-1β and TNF-*α* by competitively inhibiting the NF-κB pathway, while increasing the synthesis of Col-II and glycosaminoglycans (GAGs) (94). The combined use of the two can exert a better protective effect on cartilage (95). A clinical study showed that for patients with knee OA, supplementation with 1,500 mg of glucosamine and 1,200 mg of chondroitin for 6 months resulted in better pain relief for patients with moderate to severe pain (WOMAC ≥ 301) compared to the placebo group (*p* = 0.002), but there was no significant difference in the overall population (96). The results of a meta-analysis that included 8 RCTs (*n* = 3,793) showed that combined treatment significantly improved the WOMAC score [MD = −12.04 (−22.33 ~ −1.75); *p* = 0.02], but there was no significance in the improvement of joint space narrowing [MD = −0.09 (−0.18 ~ −0.00); *p* = 0.04] and the WOMAC stiffness score [MD = −4.70 (−8.57 ~ −0.83); *p* = 0.02] (97). Although there are still some studies that affirm the effect of the combined use of glucosamine and chondroitin in alleviating the pain of OA (98), most studies believe that the combined treatment has no value in improving the joint structure of OA patients, and there is significant heterogeneity in terms of symptom relief (99, 100). Therefore, the OARSI guidelines conditionally recommended the use of glucosamine and chondroitin for alleviating symptoms of knee OA (with evidence level B), while the ACR guidelines did not include it in their routine recommendations. Glucosamine and chondroitin have good tolerance and no serious side effects have been observed so far. However, some studies suggest that the long-term side effects need to be further investigated (101). We suggest that under the guidance of a doctor, OA patients can try taking them for 3 to 6 months. If there is no effect, the use should be discontinued. Products with high purity and standard dosage (such as crystalline glucosamine sulfate) should be preferred to optimize potential effects.

#### Oral hyaluronic acid

Oral hyaluronic acid (HA) is a form of different molecular weights (typically < 500 kDa) HA. It can be partially absorbed through the intestinal tract or metabolized by the gut microbiota into active fragments, which stimulate the synthesis of endogenous HA by synovial cells and chondrocytes, thereby increasing the viscosity of synovial fluid. At the same time, it can inhibit the TLR4/NF-κB pathway to reduce the expression of IL-6, IL-8 and COX-2, and alleviate joint inflammation and oxidative stress. It can also indirectly improve the joint microenvironment by regulating the production of SCFAs (102). The tolerance of HA is good and there are currently no reports of adverse effects (103). A 12-month RCT showed that oral HA could improve the clinical symptoms of OA patients aged 70 and below when combined with muscle strength training (104). Another RCT demonstrated that oral administration of large-molecular-weight HA can alleviate pain and improve function in patients with OA in the short term, and can also reduce the use of NSAIDs and painkillers (105). However, there are few reports on the significance in changing the thickness of cartilage. Currently, there are still many doubts about the treatment mechanisms of oral HA, so intra-articular injection of HA is more recommended in clinical practice to better exert its effect (evidence level Ib) (106).

### Probiotics

Probiotics refer to live microorganisms that, when consumed in sufficient quantities, provide benefits to the host’s health. The OA-related strains mainly come from fermented foods or commercial preparations and have the ability to resist stomach acid, bile, and establish a good colonization in the intestines. Their potential mechanisms in the prevention and treatment of osteoarthritis are as follows: (1) By restoring the balance of the intestinal flora, enhancing the integrity of the intestinal barrier, reducing LPS entry into the bloodstream, and lowering systemic low-level inflammation. (2) Inhibiting the NF-κB signaling pathway, down-regulating pro-inflammatory factors such as TNF-*α*, IL-6, and IL-1β, and reducing serum hs-CRP levels. (3) Inducing the differentiation of Treg, improving the immune microenvironment of the joint synovium, and indirectly slowing down cartilage degradation. Although the mechanism research is still ongoing, probiotics have attracted the attention of OA patients due to the “gut-joint axis” concept. Clinical studies have shown that specific strains, such as *Lactobacillus casei Shirota (LcS)*, can significantly improve the WOMAC pain score (*p* < 0.01) in patients with OA and reduce the level of hs-CRP (107). The *thermophilic Streptococcus TCI633* has shown the potential to delay the progression of OA biomarkers (such as sCTX-II), although this study has limitations due to its short duration and small sample size (108).

At present, there are several controversies in this field. Firstly, the effects of probiotics show significant strain-specificity and individual variability. Not all probiotics are effective for OA, and their efficacy largely depends on the initial intestinal flora composition of the host (109). Secondly, most of the current human studies are still at a preliminary stage. The results (such as the insignificant pain improvement in the *TCI633* study) suggest that probiotics may mainly function to delay the pathological process rather than quickly alleviate symptoms. Moreover, the long-term safety of probiotics in vulnerable populations still requires vigilance. Therefore, future research should focus on conducting large-scale, long-term, and targeted RCTs on specific OA phenotypes, with the aim of clarifying whether the effects of probiotics have disease-modifying effects. Key directions include using metagenomics to identify baseline characteristics of the gut microbiota that can predict therapeutic efficacy, developing next-generation synthetic microbiota or probiotic-prebiotic consortia for OA, and in-depth exploration of the safety of inactivated probiotics and their metabolites (postbiotics) (110). At present, it is recommended to choose strains that have evidence-based support and to combine them with a high-fiber diet to maximize their benefits.

## Dietary patterns and OA treatment

After understanding the mechanisms and evidence-based effects of key nutrients in preventing or treating OA, dietary patterns rich in beneficial nutrients have also received increasing attention. Next, we will investigate the characteristics of different dietary patterns and their potential impact on OA to assess the potential benefits and risks (Table 2).

**Table 2:** Characteristics, potential benefits and risks of different dietary patterns.

Dietary patterns	Characteristics	Effect	Precautions
Mediterranean diet	High intake of olive oil, fish, whole grains, fruits, vegetables, nuts and spices	Anti-inflammation Anti-oxidation (111–120)	Not reported
Low-calorie diet	Restrictive daily energy intake (1200–1,500 kcal)	Anti-inflammation (122) Reductive mechanical load (13)	Supplement with an appropriate amount of protein (≥1.2 g/kg/d) and combination with exercise (126)
Plant-based diet	Plant-based food such as vegetables, fruits, whole grains, beans, nuts, and seeds (127) Eliminate or strictly limit animal ingredients.	Anti-inflammation (128) Reductive mechanical load (129)	Nutrients deficiencies (131) Lack of essential amino acids (134) Increased risk of osteoporosis and fractures (137, 138) Flexible vegetarian diets were recommended. Serum vitamin B12 (131), ferritin and zinc should be monitored regularly (132)
Intermittent fasting	Alternating periods of eating and fasting (16:8 or 5:2 mode)	Metabolic regulation (139) Anti-inflammation (142, 143) Anti-oxidation (144)	Not recommended for OA patients with diabetes, eating disorders, malnutrition, sarcopenia, as well as for pregnant women and adolescents (146)

### The anti-inflammatory property of the Mediterranean diet and clinical studies

The Mediterranean Diet (MD) is characterized by high intake of olive oil, fish, whole grains, fruits, vegetables and nuts, which exerts anti-inflammatory and antioxidative effects through the synergistic effects of multiple components. The core component, extra virgin olive oil, is rich in oleocanthal, which inhibits COX-1/2 activity (similar to the mechanism of ibuprofen), and reduces the production of PGE₂ and LTB₄. In addition to oleocanthal, the MD is also abundant in various polyphenolic compounds, which confer synergistic benefits by targeting multiple pathways relevant to OA pathogenesis, including inflammation, oxidative stress, and cartilage metabolism (111–116) (Table 3). Omega-3 fatty acids (from fish) downregulate the NF-κB pathway and reduce IL-6 and TNF-*α*. Dietary fiber promotes the production of SCFAs by gut bacteria, enhancing the anti-inflammatory function of Treg cells (117, 118). Generous use of aromatic herbs and spices like garlic, ginger, and thyme further reduces systemic inflammation and oxidative stress. Clinical studies have shown that strict MD (Q5) can significantly reduce the risk and progression of OA (RR = 0.91; 95% CI: 0.82–0.998) (119). MD can significantly reduce the levels of IL-1β (~47%, *p* = 0.010) and sCOMP (1 U/L, ~8%, *p* = 0.014) in the blood of OA patients, and improve their mobility of joint (*p* < 0.05) (120). When compared with a low-fat diet, MD could better alleviate the pain of OA patients (*p* = 0.04), which indicates the unique anti-inflammatory property of MD that distinguishes it from general weight loss diets. Considering that weight loss remains the first-line treatment for OA, especially for obese patients (121), and MD, as a basic dietary pattern, can simultaneously achieve weight loss and anti-inflammatory effects, thus, it has certain therapeutic value for OA patients.

**Table 3:** Key polyphenols in the Mediterranean diet and their potential roles in OA management.

Polyphenol compound	Primary dietary sources in MD	Mechanisms of action	Level of evidence
Oleocanthal	Extra virgin olive oil	Non-selectively inhibits COX-1 and COX-2, reducing pro-inflammatory prostaglandins such as PGE2 (111)	Strong preclinical
Hydroxycinnamic Acid	Whole grains, coffee, artichokes, herbs	Experimental treatment suppressed H₂O₂-induced expression of IL-1β, TNF-*α*, MMP-1, and MMP-13, while increasing SOX9 gene expression (112)	Strong preclinical
Resveratrol	Red grapes, red wine, peanuts, berries	SIRT1 activation inhibits NF-κB via deacetylation, upregulates Nrf2/HO-1 axis, and induces autophagy (113)	Strong preclinical
Anthocyanins	Berries (blueberries, strawberries), eggplants, red onions	Suppresses IL-1β-induced NF-κB/iNOS activation and scavenges ROS to mitigate oxidative stress (114)	Strong preclinical
Quercetin	Capers, onions, kale, apples	Dual COX-2/5-LOX inhibition suppresses prostaglandin/leukotriene production, downregulates NF-κB/MAPK pathways, and attenuates cartilage degradation in arthritis models (115)	Strong preclinical
Lycopene	Tomatoes, watermelon, cooked tomato products	A potent carotenoid that quenches singlet oxygen and is linked to reduced systemic inflammation (116)	Strong preclinical

However, a key counterargument is that the observed benefits in observational studies may be confounded by the overall healthier lifestyle of MD adherents, who typically exhibit higher physical activity levels and lower rates of smoking. Furthermore, the long-term sustainability of the MD in non-Mediterranean populations poses a significant challenge for widespread implementation, and the specific contribution of its individual components remains difficult to isolate. Therefore, future research should prioritize long-term, randomized controlled trials designed to disentangle the effects of the MD from other healthy behaviors. Key directions include investigating the diet’s impact on structural disease progression using advanced imaging and exploring culturally adapted, supported behavioral interventions to enhance adherence and translate this promising dietary pattern into real-world clinical practice for diverse OA populations. Even more, by conducting a detailed analysis of the effects and proportions of each component in the MD diet, we can make specialized adjustments for different patients to balance the therapeutic effect and the acceptance.

### The efficacy of low-calorie diets on obesity-related OA

Low-Calorie Diet (LCD) induces weight loss by restricting daily energy intake (typically 1,200–1,500 kcal), which plays a dual protective role in obesity-related OA. (1) It reduces mechanical load, thereby decreasing cartilage wear and abnormal stress on subchondral bone (13). (2) It regulates metabolic inflammation. The reduction of visceral fat down-regulates pro-inflammatory adipokines (leptin, resistin), up-regulates anti-inflammatory adiponectin, and inhibits the NF-κB pathway and the release of IL-1β and TNF-*α* in synovial macrophages. Simultaneously, it improves insulin sensitivity and reduces the damage of AGEs to cartilage (122). The IDEA (Intensive Diet and Exercise for Arthritis) trial (*n* = 454) demonstrated that obese patients with knee OA who received diet-related weight loss combined with exercise for 18 months showed significant improvements in pain (*p* < 0.05) and function (*p* < 0.005) (123). In addition to the symptoms improvements, clinical studies have shown that weight loss induced by a low-calorie diet can increase the concentration of adiponectin in the serum (*p* = 0.0480) and decrease the concentration of COMP (*p* < 0.0001) (124), which indicated a lower risk of cartilage damage. From an imaging perspective, weight loss was observed to reduce the number of pixels in the cartilage layer on T2 images (*p* = 0.001), which was associated with the alleviation of pain (*p* = 0.02) and function (*p* = 0.03). A weight loss of more than 10% significantly inhibited the progression of OA over the 48-month follow-up period (125). In conclusion, low-calorie diets and weight loss have a good therapeutic effect and have been classified as strong recommended treatments for obesity-related OA. However, the counterargument lies in the challenge of long-term sustainability. While efficacy is clear in controlled trials, real-world adherence to stringent calorie restriction is often poor, with high rates of weight regain. This raises questions about the practicality of LCD as a standalone long-term strategy for most individuals. Furthermore, weight loss alone may not fully resolve the dysregulated metabolic and inflammatory milieu in all patients, particularly those with longstanding OA, indicating the need for complementary anti-inflammatory interventions.

Therefore, future research should pivot toward sustainable dietary patterns that incorporate LCD principles but focus on food quality and satiety (e.g., MD) to enhance long-term adherence. Key directions include investigating the role of body composition changes (preserving muscle mass during weight loss) on clinical outcomes and developing personalized nutritional approaches based on metabolic phenotypes to identify those most likely to benefit from specific dietary interventions. When choosing a low-calorie diet, it is important to supplement an appropriate amount of protein [≥1.2 g/kg/d (126)] and combine functional exercises to maintain muscle strength.

### The potential benefits and risks of a vegetarian diet

Vegetarianism (or plant-based diet) is a dietary pattern that mainly consists of foods derived from plants, excluding or strictly limiting animal ingredients such as meat, poultry, and fish. The core foods include vegetables, fruits, whole grains, beans, nuts, and seeds (127). The high intake of fruits, vegetables, legumes and nuts in a vegetarian diet provides abundant polyphenols (such as quercetin) and vitamin C, which inhibit the NF-κB pathway and COX-2 activity, and reduce inflammatory markers such as IL-6 and CRP (128). Meanwhile, the low energy density diet represented by vegetarianism helps with weight loss and reduces the mechanical load on joints (129). A vegetarian diet also reduces the intake of AGEs (such as grilled meats) and minimizes the damage to cartilage caused by the AGEs-RAGE axis (130). Although there are certain benefits, the vegetarian pattern still has some risks and controversies. Firstly, long-term vegetarianism can lead to nutrients deficiencies. Among them, vitamin B12 deficiency causes an increase in homocysteine levels (131), which may exacerbate oxidative stress and apoptosis of chondrocyte. The absorption rate of zinc combined with phytate and plant-based non-heme iron is low, which may weaken the activities of SOD/GPx (132). Strict vegetarians may suffer from EPA/DHA deficiency (133), and they need to rely on the conversion of ALA, which might affect the anti-inflammatory effect of *ω*-3 PUFAs. Secondly, the lack of essential amino acids (such as lysine) in plant proteins may reduce the synthesis of cartilage matrix (134). Currently, multiple RCT studies have shown that the vegetarian diet can significantly improve the WOMAC scores (*p* ≤ 0.0001) and reduce inflammatory indices in the short term (from 4 months to 1 year) (135, 136). However, long-term follow-up studies have revealed that this dietary pattern increased the risk of osteoporosis and fractures (137, 138), which is likely to accelerate the progression of OA. Therefore, it is recommended to adopt an elastic vegetarian diet (such as a diet mainly consisting of plants with the addition of fish, eggs, and dairy products) for the management of OA, and avoid strict vegetarianism. At the same time, regular monitoring of serum B12, ferritin and zinc levels should be conducted, and supplementation should be provided when necessary.

### The regulatory effect of intermittent fasting on metabolism and inflammation

Intermittent Fasting (IF) is a periodic energy intake restriction pattern, which includes alternate eating (5:2 pattern, restricting calorie intake to 500–600 kcal on 2 days per week) and fasting periods (such as 16:8 pattern, fasting for 16 h each day). IF may exert therapeutic effects through the following three mechanisms. One is metabolic regulation. During fasting periods, insulin secretion is reduced, the AMPK pathway is activated, and glucose uptake and fatty acid oxidation are increased, which will improve insulin resistance and obesity-related OA (139). At the same time, fasting induces the expression of key genes for autophagy (ATG5/LC3), removes damaged mitochondria and misfolded proteins, and inhibits apoptosis of chondrocytes (140, 141). The second effect is anti-inflammation. Beyond reducing visceral fat and suppressing the NF-κB pathway (142), emerging evidence suggests IF may directly inhibit the activation of the NLRP3 inflammasome, a key sensor of metabolic stress that drives the production of IL-1β (143). Thirdly, by enhancing the activity of antioxidant enzymes (SOD, GPx), IF can reduce the generation of ROS in mitochondria and block the oxidative cascade reaction (144). At present, there are not many reports on the use of IF in the treatment of OA (145). Therefore, the actual efficacy and long-term safety are not definite. However, some studies have shown that for patients with type II diabetes and a history of taking antidiabetic medications, the use of IF should be avoided, as there is a risk of causing hypoglycemia (146). In a word, it is not recommended to use IF to achieve weight control and OA treatment, especially for patients with diabetes, eating disorders, malnutrition, sarcopenia, pregnancy, and adolescent OA.

## Nutrition management for special OA populations

Although many nutrients and dietary patterns can exert certain therapeutic or preventive effects on OA by regulating metabolism, inflammation and oxidation, and maintaining cartilage homeostasis, the application of them still requires individualized adjustments to avoid the occurrence of other nutrition-related adverse events. Therefore, in the following content, we will provide customized nutrition management strategies for special OA populations.

### Nutritional management for elderly patients with OA

**Figure 3:**
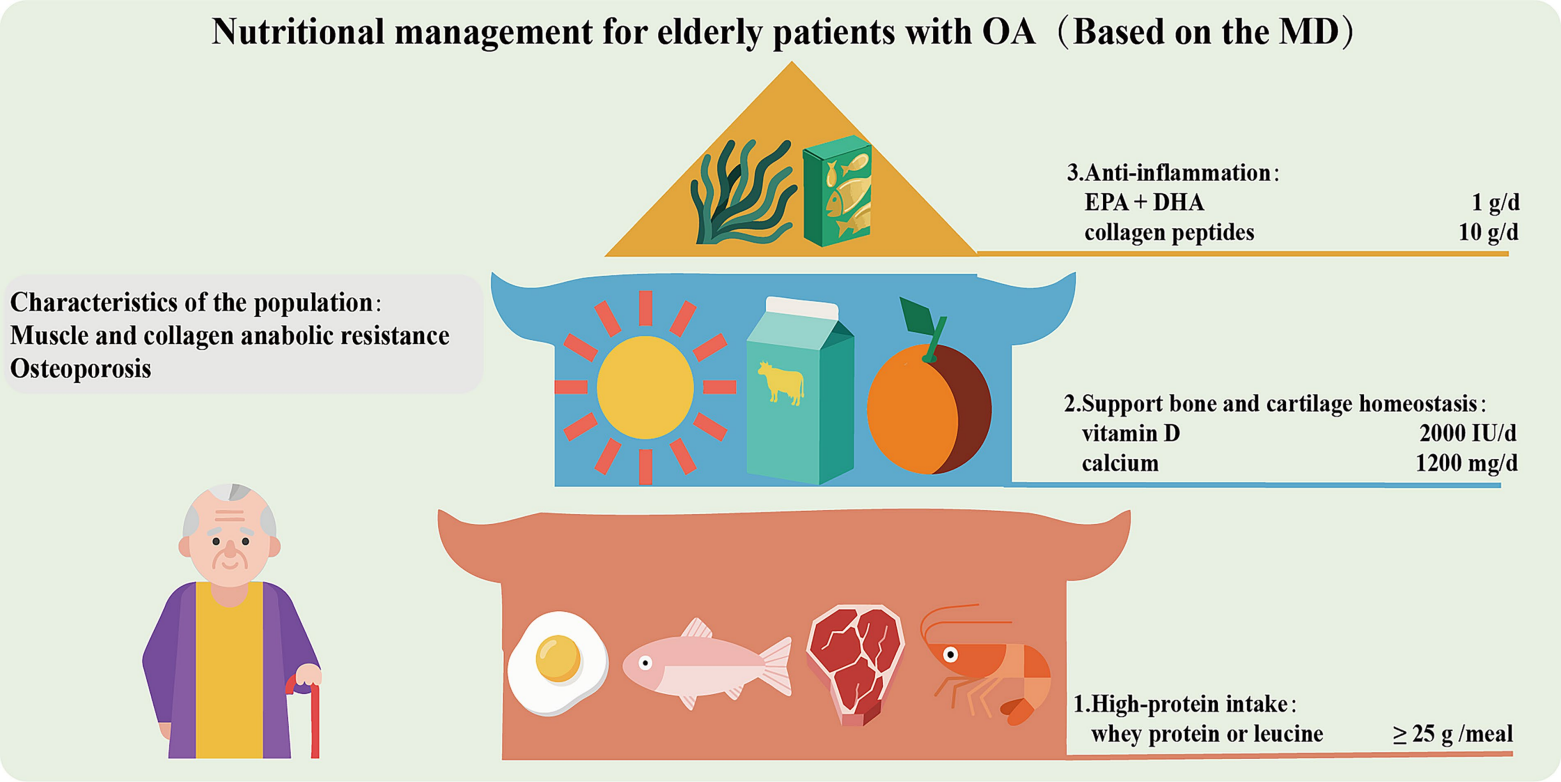
Nutritional management for elderly patients with OA.

In elderly patients with OA, the decline in growth hormone and insulin-like growth factor 1, and the impaired differentiation of osteoblasts lead to resistance in muscle and collagen synthesis, and osteoporosis (147). The frequent pain and limited mobility lead to a significant reduction in physical activity, resulting in long-term deficiencies of vitamin D and calcium. Moreover, the reduced mechanical load stimulation and the use of NSAIDs further inhibit the synthesis of muscle protein and collagen (148), and exacerbate the destruction of bone trabecular microstructure. The above pathological factors necessitate that the nutritional strategy for elderly patients with OA should focus on anti-inflammation, muscle protection and metabolic regulation (Figure 3). Firstly, it is emphasized that high protein intake (1.2–1.5 g/kg/d) is of great importance (149). Prioritize the intermittent supplementation of whey protein or foods rich in leucine (such as eggs and fish), with each meal containing at least 25 grams to promote muscle synthesis and prevent sarcopenia, which will help prevent the progression of OA. Secondly, it is necessary to enhance the intake of vitamin D (2000 IU/day) and calcium (1,200 mg/day) to maintain serum 25(OH)D_3_ more than 30 ng/mL, which will support the stability of bone and cartilage. Additionally, *ω*-3 PUFAs (EPA + DHA 1 g/day) and collagen peptides (10 g/day) are also recommended to promote cartilage matrix synthesis. Overall, the MD (≥ 30 mL of extra virgin olive oil per day and ≥ 2 servings of fish per week) should be the basic diet for elderly patients with OA. High AGEs diets (such as grilled red meat) should be restricted. Calorie intake should be more than 30 kcal/kg/day. Obese patients should moderately limit calories (~500 kcal/day), but the total should not be too low to avoid malnutrition. Regular monitoring of renal function (eGFR) and muscle strength (grip strength or walking speed) is necessary. Patients with advanced kidney disease should adjust protein intake (150).

### Blood glucose control and nutritional balance in OA patients with diabetes

**Figure 4:**
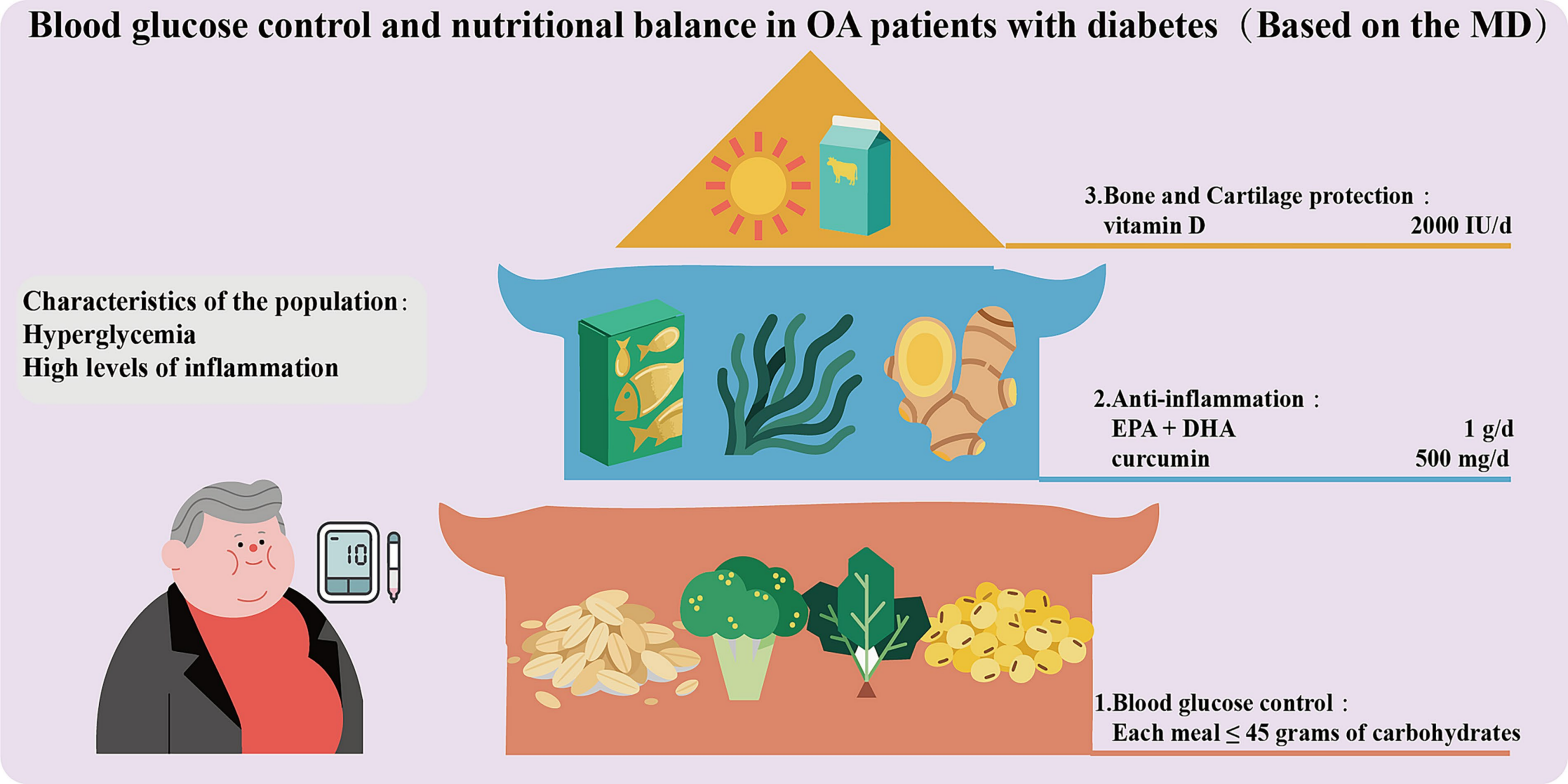
Blood glucose control and nutritional balance in OA patients with diabetes.

When OA patients are combined with diabetes, high blood glucose can lead to the accumulation of AGEs (151), which will activate RAGE-dependent inflammatory pathways and disrupt the stability of the cartilage matrix, thereby accelerating the progression of OA. Therefore, these patients need to simultaneously regulate blood glucose and joint inflammation through precise nutritional strategies (Figure 4). Blood glucose control should be given top priority. A low glycemic index (GI) diet consisting of whole grains (oats, quinoa), legumes, and non-starchy vegetables (broccoli, spinach) should be adopted as the main dietary structure. Refined carbohydrates (with a GI > 70) should be restricted to maintain post-meal blood glucose below 7.8 mmol/L (152). Each meal should contain no more than 45 grams of carbohydrates (accounting for 40% of total calories), which should be evenly distributed across the three meals to prevent excessive fluctuations in blood glucose and increased oxidative stress (153). *ω*-3 PUFAs (EPA + DHA 1 g/day) and polyphenols (curcumin 500 mg/day + piperine) should be recommended to achieve anti-inflammatory effects and improve insulin sensitivity (154). Furthermore, vitamin D (2000 IU per day) can also be appropriately supplemented to protect the cartilage and reduce the risk of joint damage related to diabetic neuropathy (155). Overall, the MD is also applicable to OA patients combined with diabetes. Such patients should avoid foods with high AGEs and strictly limit fructose (such as sugary beverages). HbA1c, vitamin D, and urine microalbumin should be tested every 3 months to monitor for diabetic kidney disease.

### Joint nutrition support for OA caused by physical activity

**Figure 5:**
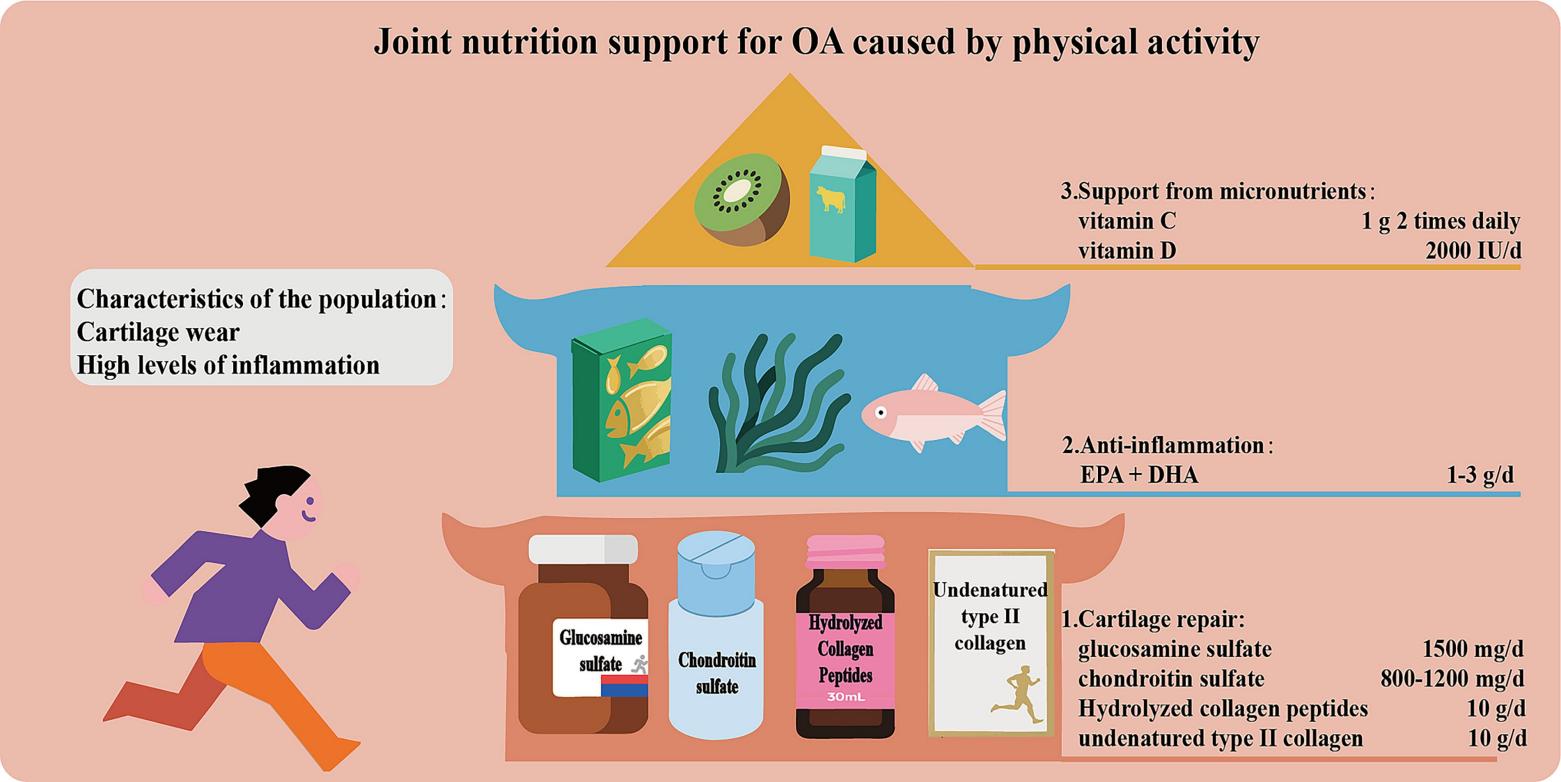
Joint nutrition support for OA caused by physical activity.

Due to increased joint load, people who are physically active are prone to cartilage wear and inflammation. Therefore, scientific and reasonable support for cartilage nutrition is of vital importance. The core strategies include the following (Figure 5): (1) Supplement with glucosamine sulfate (1,500 mg/day) and chondroitin sulfate (800–1200 mg/day) as precursors of cartilage matrix to promote cartilage repair. Intake of hydrolyzed collagen peptide (especially Col-II, 10 g/day) or non-denatured Col-II stimulates collagen synthesis and regulates immune response. (2) Supplement with *ω*-3 PUFAs (EPA + DHA 1–3 g/day) to inhibit the production of pro-inflammatory factors. (3) Ensure the levels of micro-nutrients such as vitamin C and D to support collagen synthesis and bone metabolism.

### Nutritional management of OA patients in perimenopausal and postmenopausal women

**Figure 6:**
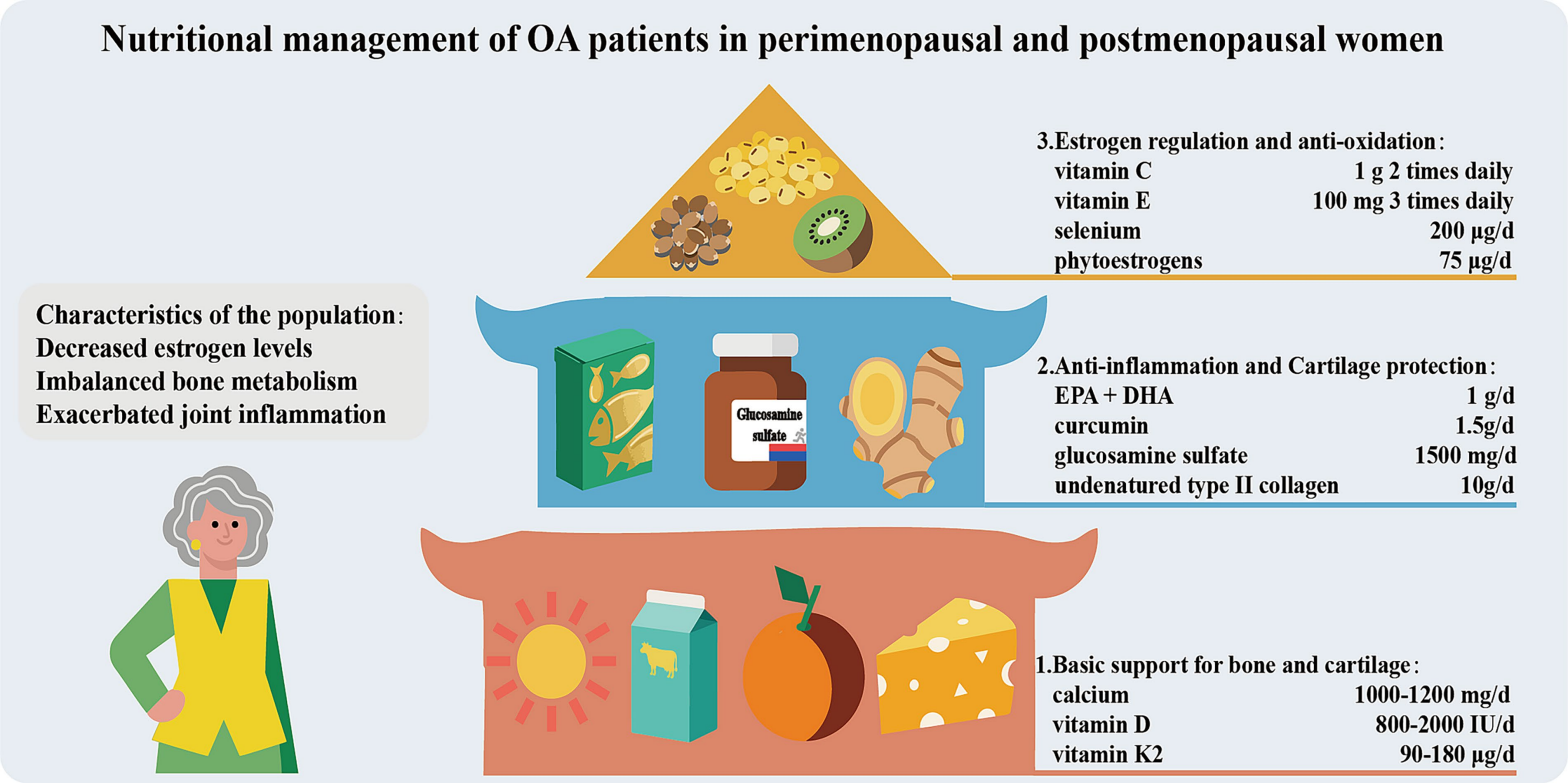
Nutritional management of OA patients in perimenopausal and postmenopausal women.

Perimenopausal and postmenopausal women experience a significant decline in estrogen levels, resulting in bone metabolism imbalance (bone resorption > bone formation) and exacerbated joint inflammation. Consequently, the risk of OA increase and symptoms worsen. Therefore, nutritional management should be carried out in three ways (Figure 6). Firstly, basic support for bones and joints should be provided. Adequate calcium (1000–1,200 mg/day, taken in divided doses) and vitamin D (800–2000 IU/day, maintaining blood 25(OH)D_3_ > 30 ng/mL) intake is the core for maintaining bone density and reducing the risk of fractures (156). Vitamin K2 (MK-7, 90–180 μg per day) can promote the carboxylation of osteocalcin, guiding calcium to be deposited in the bones rather than in soft tissues (157). Secondly, anti-inflammation and cartilage protection should be focused on. Supplementing with *ω*-3 PUFAs and curcumin can alleviate joint inflammation. Non-denatured Col-II and glucosamine sulfate promote the synthesis of cartilage matrix. Additionally, increasing the intake of foods rich in antioxidants (vitamin C, E, selenium) and phytoestrogens (soy isoflavones, flaxseed lignans) can assist in alleviating menopausal symptoms and oxidative stress damage. Strictly controlling body weight (BMI < 25) to reduce joint load is of crucial importance.

## Practical suggestions and future perspectives

**Figure 7:**
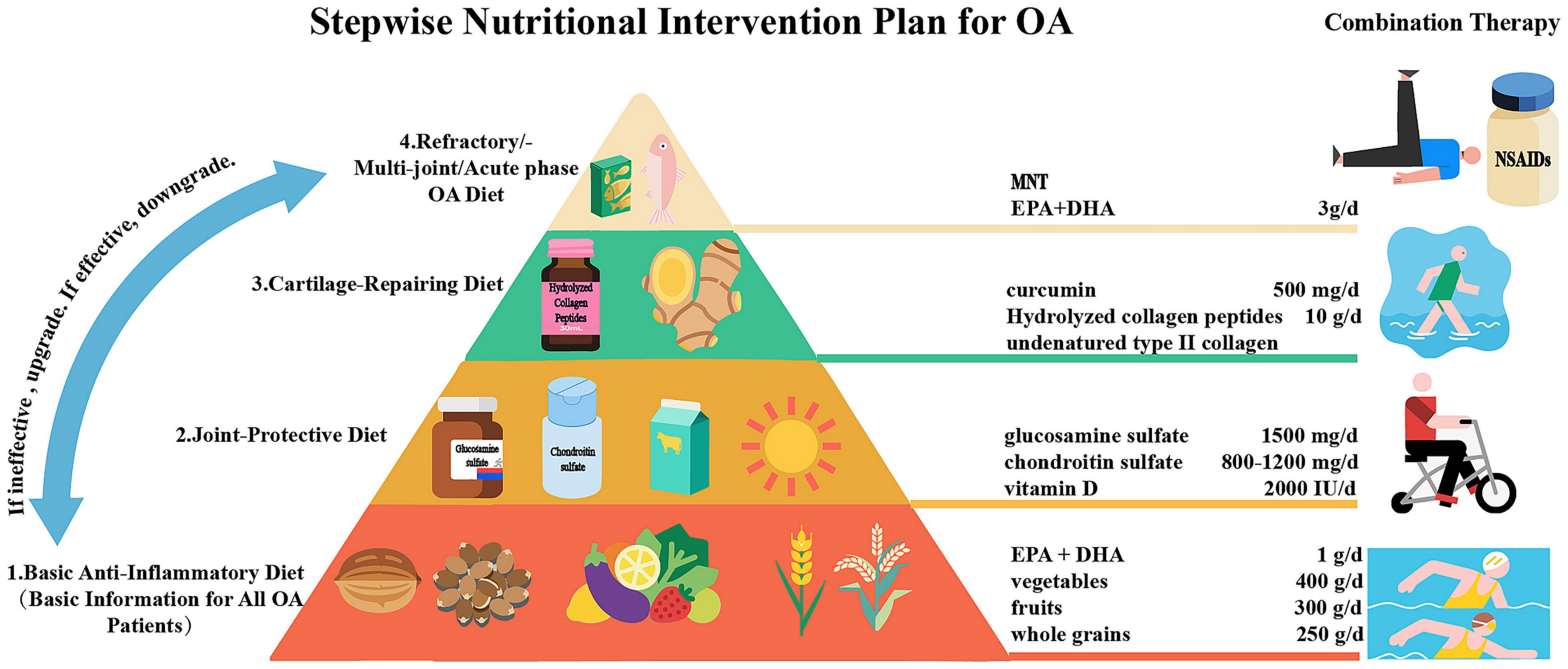
A proposed stepwise nutritional intervention plan for OA patients to relieve pain, delay cartilage degeneration, improve function, and reduce the need for drugs and surgical intervention.

After in-depth exploration of nutritional intervention for OA, we have developed a stepwise nutritional intervention plan (Figure 7) based on evidence from clinical studies. This plan aims to relieve pain, delay cartilage degeneration, improve function, and reduce the need for drugs and surgical intervention. It is divided into four progressive steps. The first step (for all OA patients) is following an anti-inflammatory diet (mainly the MD, rich in *ω*-3 PUFAs, fruits, vegetables, and whole grains), combined with weight loss of 5–10% (for those with a BMI ≥ 25) and low-impact exercises (such as swimming) for 3 months. If the VAS score reduction is less than 20%, move to the next step. The second step is adding joint structure support nutrients, including glucosamine sulfate, chondroitin sulfate, and vitamins D3 and K2. The third step (for insufficient response to the second step) is introducing high-activity cartilage repair components, such as non-denatured Col-II and hydrolyzed collagen peptides (10 g/day). Curcumin should be used to inhibit inflammatory pathways. The fourth step (for refractory/multi-joint OA) is integrate medical nutrition therapy (MNT) based on the previous steps, combined with high-dose ω-3 PUFAs (EPA + DHA 3 g/day) for enhanced anti-inflammation. This step should be based on joint fluid biomarkers (such as COMP, CTX-II), and coordinated with physical therapy (strength training, low-frequency pulsed current stimulation, cold and heat therapy, etc.) and drugs (such as NSAIDs, intra-articular injections). Each step lasts for 3 months. Pain scores, joint function (WOMAC index), and inflammatory markers (hs-CRP) should be assessed dynamically. If effective, maintain or downgrade the step. If ineffective or progressing, upgrade. Liver and kidney function and gastrointestinal tolerance should be monitored throughout the process.

In addition to the stepwise nutritional treatment plan, we have also extended nutritional intervention to the prevention of OA. Primary prevention strategies for OA should target modifiable dietary risk factors that drive obesity and metabolic dysfunction, key contributors to disease onset. These factors predominantly include the frequent consumption of sugar-sweetened beverages, ultra-processed foods, and diets high in saturated fats, which promote weight gain and chronic low-grade inflammation (158). Conversely, emphasizing a pre-emptive anti-inflammatory dietary pattern from early adulthood, such as MD, is advocated. It is also advisable to supplement multivitamins (such as VD, VC, VE, etc.), calcium and probiotics (*LcS*) in a preventive manner. However, it is not recommended to take them for a long time or in excessive amounts to avoid adverse reactions. These approaches focus on maintaining a healthy weight and mitigating systemic inflammation before the clinical onset of OA. Public health initiatives promoting these approaches, alongside reduced intake of pro-inflammatory foods, represent a cost-effective strategy to lower the population-wide burden of obesity-related OA.

Despite the promising evidence supporting nutritional interventions for OA, several critical limitations in the current research landscape must be acknowledged to contextualize these findings. First, there is considerable heterogeneity in study designs, including wide variations in the dosage, bioavailability, and treatment duration of supplements (e.g., *ω*-3 PUFAs, collagen peptides), which complicates direct comparison and meta-analysis. Second, many clinical trials are of relatively short duration (often ≤6 months), failing to capture the long-term efficacy and sustainability of interventions on OA progression, a inherently chronic disease. Furthermore, most studies prioritize symptom improvement (e.g., pain reduction) as endpoints, with a pronounced lack of robust evidence demonstrating structural modification (e.g., cartilage preservation via quantitative MRI). The generalizability of findings is also often limited by insufficient consideration of individual differences, such as genetic background, baseline nutritional status, gut microbiota composition, and specific OA phenotypes (e.g., metabolic vs. post-traumatic). Finally, the majority of mechanistic insights are derived from preclinical models, and the translation of these pathways to human pathophysiology remains incompletely elucidated. Addressing these limitations through standardized, long-term, and phenotype-specific randomized controlled trials is imperative for advancing the field of nutritional OA.

We support the importance of a multidisciplinary collaboration model (orthopedics, nutritionists, rheumatology, rehabilitation) in the treatment of OA. Although physical therapy and drug management still dominate OA treatment, the establishment of a stepwise nutritional intervention plan is believed to assist in delaying OA progression, reducing drug use, and avoiding surgical risks. In the future, with the advancement of digital health technology, multi-dimensional data integration and intelligent decision-making will make nutritional intervention more intelligent, customized, and refined. At the same time, with the in-depth research on microbiome-targeted therapy and nutritional epigenetics, the exploration of beneficial and non-beneficial nutrients will be more comprehensive, and the efficiency of nutritional intervention will significantly increase. Nutritional intervention is expected to become the third major treatment modality alongside physical therapy and drug therapy, bringing significant benefits to patients with mild to moderate OA and effectively reducing the proportion of severe OA patients, which allow more people to avoid the adverse effects of repeated drug treatment and the potential risks of surgery and prosthesis replacement.

## Conclusion

This review elucidated the significance of the metabolism-inflammation-oxidative stress axis in the occurrence and development of OA, and also dialectically discussed the mechanisms and clinical benefits of beneficial nutrients in the treatment of OA. Among them, *ω*-3 PUFAs (EPA and DHA) and polyphenols (curcumin) are regarded as key nutrients with potent anti-inflammatory effects, and also the main effect components in the recommended dietary pattern, MD. Collagen peptides, vitamin D, calcium, probiotics, glucosamine and chondroitin are believed to be suitable for supplementation in moderation for the treatment and prevention of OA. By analyzing and comparing the risks and benefits of different dietary patterns, we have developed a stepwise nutritional intervention strategy based on MD for the auxiliary treatment of different types of OA patients and emphasized the importance of comprehensive intervention (physics and medication) in the treatment of OA. Even more, we have specifically tailored the nutritional intervention based on the characteristics of different groups of people. This review will provide new treatment ideas for a large number of OA patients, while avoiding the abuse of drugs and early artificial joint replacement surgeries. It also provides nutritional suggestions and evidence-based data for the prevention of OA. Although there are still some limitations in the current research, we believe that in the future, systematic nutritional intervention is expected to become the third major treatment alongside physical and drug therapy, benefiting more people.
